# The role of neuromuscular ultrasound in diagnostics of peripheral neuropathies induced by cytostatic agents or immunotherapies

**DOI:** 10.1186/s40478-023-01685-9

**Published:** 2023-11-27

**Authors:** Stefanie Hartinger, Jakob Hammersen, Niklas A. Leistner, Anna Lawson McLean, Clemens Risse, Christian Senft, Stefanie Schütze, Bianka Heiling, Matthias Schwab, Irina Mäurer

**Affiliations:** 1 Department of Neurology, Jena University Hospital, Friedrich Schiller University Jena, Am Klinikum 1, 07747 Jena, Germany; 2 Neurooncological Center, Jena University Hospital, Friedrich Schiller University Jena, Jena, Germany; 3 Klinik für Innere Medizin II, Hämatologie, Internistische Onkologie und Palliativmedizin, Universitätsklinikum Jena, Friedrich-Schiller-Universität Jena, Jena, Deutschland; 4 Department of Neurosurgery, Jena University Hospital, Friedrich Schiller University Jena, Jena, Germany; 5 Department of Gynecology and Reproductive Medicine, Jena University Hospital, Friedrich Schiller University Jena, Jena, Germany

**Keywords:** Neuromuscular ultrasound, Cancer, Peripheral neuropathy, Chemotherapy, Immunotherapy, Neurotoxicity

## Abstract

A relevant number of cancer patients who receive potentially neurotoxic cytostatic agents develop a chemotherapy-induced peripheral neuropathy over time. Moreover, the increasing use of immunotherapies and targeted agents leads to a raising awareness of treatment-associated peripheral neurotoxicity, e.g., axonal and demyelinating neuropathies such as Guillain–Barré-like syndromes. To date, the differentiation of these phenomena from concurrent neurological co-morbidities or (para-)neoplastic nerve affection as well as their longitudinal monitoring remain challenging. Neuromuscular ultrasound (NMUS) is an established diagnostic tool for peripheral neuropathies. Performed by specialized neurologists, it completes clinical and neurophysiological diagnostics especially in differentiation of axonal and demyelinating neuropathies. No generally approved biomarkers of treatment-induced peripheral neurotoxicity have been established so far. NMUS might significantly extend the repertoire of diagnostic and neuromonitoring methods in this growing patient group in short term. In this article, we present enlargements of the dorsal roots both in cytostatic and in immunotherapy-induced neurotoxicity for the first time. We discuss related literature regarding new integrative applications of NMUS for cancer patients by reference to two representative case studies. Moreover, we demonstrate the integration of NMUS in a diagnostic algorithm for suspected peripheral neurotoxicity independently of a certain cancer treatment regimen emphasizing the emerging potential of NMUS for clinical routine in this interdisciplinary field and prospective clinical trials.

**Supplementary Information:**

The online version contains supplementary material available at 10.1186/s40478-023-01685-9.

## Introduction

### Paraclinical assessment of neuropathies

In general, neurological examination and electrodiagnostic studies are the gold standard in the diagnosis of (poly-)neuropathies. Thereby, electrodiagnostic studies reveal either a sensory neuronopathy affecting dorsal root ganglia with a diffuse decrease of amplitudes or even complete lack of sensory nerve action potentials (SNAP) and normal sensory conduction velocities or a distal, dying-back, axonal degeneration with reduction of sensory action amplitudes starting in the legs and then progressing to the arms [1]. Primary demyelination with slowing of motor conduction or F-wave abnormalities (F-waves are a late response following the motor response and is evoked by supramaximal electrical stimulation of a mixes or motor nerve) [2] as well as motor involvement with reduced compound muscle action potential amplitudes and evidence of denervation changes in electromyography are seen less frequently. A reduction in the amplitude of compound muscle action potentials (CMAPs) and SNAP with only a slight decrease of conduction velocity (< 20%) is characteristic of axonal polyneuropathies. In contrast, demyelinating polyneuropathies show reduced motor and sensory nerve conduction velocities (> 20%), increased temporal dispersion of CMAPs, increased distal motor latencies (> 20%) and prolonged F-wave latencies [3]. A combined occurrence of the typical findings of axonal and demyelinating polyneuropathies frequently impedes a clear distinction in this patient group. At the same time, this differentiation is the crucial point in the precise diagnosis of polyneuropathies regarding aetiology and optimal treatment (e.g., immunosuppressive agents in case of inflammatory demyelinating conditions). Nerve biopsy as a comparatively invasive procedure may be discussed in individual cases if relevant for further patient care management.

In the neurological setting, high-resolution ultrasound (HRUS) of nerves and muscles (= NMUS) has become an important non-invasive tool in differential diagnosis as it is widely used in the evaluation of suspected (poly-)neuropathies [4]. The most common abnormality of interest in NMUS is nerve enlargement [4]. Diffuse enlargement of multiple locations can be detected in inflammatory neuropathies such as chronic inflammatory demyelinating polyneuropathy (CIDP) or Guillain–Barré syndrome (GBS). More focal nerve enlargement occurs in peripheral nerve tumors or compression syndromes, e.g., carpal-tunnel-syndrome. Furthermore, the assessment of nerve echogenicity and vascularity may be considered, although only few studies discuss hyper-vascularization as a marker for disease activity in CIDP [5]. Several methods have been applied to describe the pattern and extent of nerve enlargement [4].

We sought to employ the Ultrasound Pattern Sum Score (UPSS; consisting of three sub-scores (UPS-A, -B and -C)), which quantifies nerve enlargement through a weighted rating system scoring the presence of nerve swelling and swelling degree at different sites and can be used to differentiate between acute and subacute or axonal and demyelinating neuropathies [6].

First prospective studies use NMUS outside of the typical applications, e.g., for the characterization of diabetic neuropathy by a research group from our institution [7]. NMUS also has been shown to be a reliable tool in the assessment of peripheral nerve tumors including subclinical manifestations. Especially in the context of phacomatosis where regular follow-up of peripheral nerve tumors is required, NMUS is an easily accessible and inexpensive tool for screening and follow-up [8, 9]. Winter et al. showed that there were significant differences in cross sectional area (CSA) of peripheral nerves, vagus nerve and cervical roots between neurofibromatosis type 1 (NF1) patients, patients with *nf2*-related schwannomatosis (formerly known as neurofibromatosis type 2 (NF2)) and healthy controls. In addition, they found distinct ultrasound patterns for frequent plexiform neurofibromas in NF1 and mutilocular schwannomas in NF2 [10]. NF1, NF2 and schwannomatosis are rare tumor predisposition syndromes which are characterized by tumors of the central and peripheral nervous system [11]*.*

For cancer patient care, there are no general NMUS assessment recommendations.

### Cancer treatment-induced neuropathies

Chemotherapy-induced peripheral neuropathy (CIPN) is a relevant dose-limiting toxicity of several anticancer treatment regimens. In most cases, CIPN presents as a sensory neuropathy because of the damage to large-diameter sensory myelinated (Aβ) fibres or dorsal root ganglia [1]. On the other hand symptoms of CIPN may include pain, temperature disturbances and autonomic dysregulation (see Table 1 at Kandula et al. [12]), which is an indication that thin myelinated (Aδ) and unmyelinated (C) fibres are also affected.

Platinum compounds cause cell cycle arrest and apoptotic cell death by binding to cellular DNA [13]. The associated loss of dorsal root ganglia cells leads to a sensory neuronopathy (non-length-dependent) with anterograde neuronal degeneration, which might be permanent. Further pathogenic mechanisms of platin derivates such as abnormal kinetics of voltage-gated Na+ channels in the dorsal root ganglia cells seem reversible as many patients recover partly from CIPN over time [1]. In chronic administration. Oxaliplatin causes a different pattern of excitability change with abnormalities in sensory axons [14]. Moreover, greater excitability changes were seen in patients with severe neurotoxicity after treatment completion compared to those with mild or moderate neurotoxicity [14]. Another underlying pathology is assumed to be based on pro-inflammatory effects in the context of oxidative stress and mitochondrial dysfunction accompanied by production of cytokines as interleukin 1b and tumor necrosis factor [15]. Being potentially produced by microglial and neuronal cells the pro-inflammatory cytokines may affect dorsal horn neurons involved in neuropathic pain control [15].

Treatment with taxanes, vinca alkaloids and bortezomib cause defects in axonal transport by affecting microtubules which leads to a length-dependent, predominantly sensory, axonal polyneuropathy [1].

The side effects of modern antineoplastic therapies such as immunotherapies (e.g., immune checkpoint inhibitors) on the peripheral and central nervous system are predominantly driven by inflammatory processes [16]. The spectrum of these neurological pathologies includes immune-related polyneuropathies, Guillain–Barré syndrome-like conditions, myasthenia gravis, myopathy, myositis, posterior reversible encephalopathy syndromes, meningitis and encephalitis. In patients treated with immune checkpoint inhibitors, neuromuscular immune-related adverse events (irAEs) are the most common neurological complication. Consequently, peripheral nerve involvement may appear as cranial neuropathy, axonal and demyelinating peripheral neuropathy, small fiber and autonomic neuropathy [17].

Neurological irAEs (nirAEs) are described to be a relatively rare condition compared to effects on other organ systems in the context of colitis, hepatitis, endocrinopathies and pneumonitis [8]. However, the number of prospective studies focusing on neurological side effects of immunotherapies is encouragingly growing, providing us with new insights into previously accepted paradigms. Thus, currently published cross-sectional study results by Möhn et al. demonstrated the increased frequency (32.7% of 110 patients) of nirAEs in a prospective monocentric cancer patient cohort. Beyond that, the authors describe sensory deficits and manifest neuropathies in 47% and 28% of the nirAE cases, respectively [9]. The data support our clinical experience showing a much more frequent subclinical peripheral nervous system (PNS) involvement than has been previously described in the literature for patients treated with immune checkpoint inhibitors. The peripheral nerve affection may accompany the superficial central nervous system (CNS) neurotoxicity or systemic inflammation. The relevance of the slight/subclinical PNS inflammation and the role of additional assessments by specialized neurologists for the long-term outcome of affected cancer patients is still unclear.

In the context of neurological irAEs (nirAEs), previous case studies reported MRI pathologies including contrast enhancement of nerves and dorsal roots in immunotherapy-induced GBS and CIDP [18–20]. Currently, the integration of NMUS in the neurological differential diagnosis of nirAE, corresponding to distinction between demyelinating and axonal neuropathies, has not yet become a part of international consensus standards.

As Möhn et al. [9] discuss in their current work, the relatively increase in the incidence of irAE-associated neuropathies and other neurological phenomena may occur due to neurological assessments directly integrated in oncological patient care. The definite appraisal of the effects of interdisciplinary assessment for nirAE in treatment management and outcome will only be possible once there have been dedicated prospective trials focusing on characterization of immune-related neurotoxicity.

## Clinical application of NMUS in two representative case studies

**Fig. 1:**
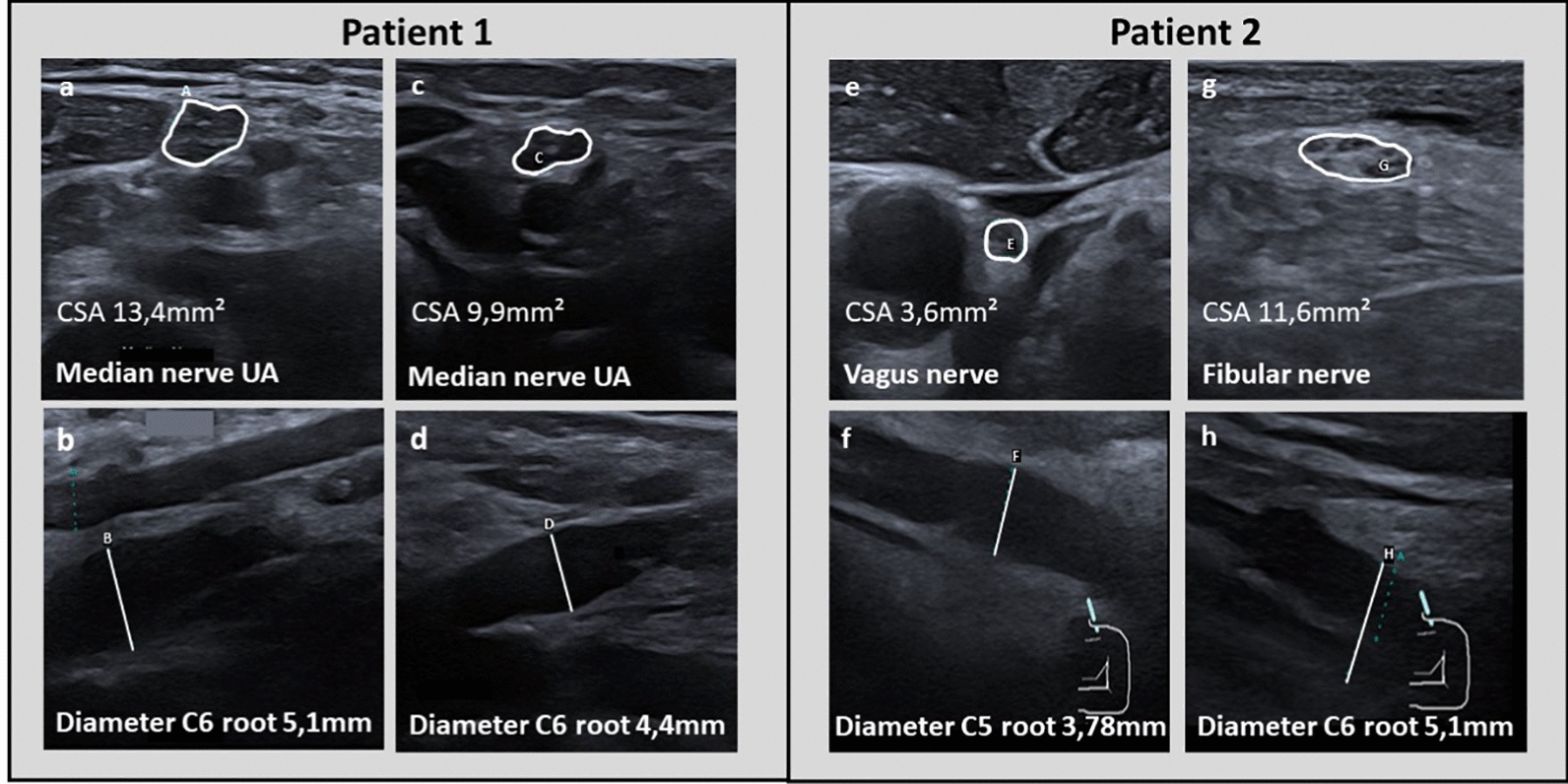
Representative nerve-ultrasound results from two case studies. The white bordered areas display the perimeter measurement of the nerves used for CSA calculation. Diameter measurements of the described nerves are marked by the straight white lines. Patient 1: NMUS showed an increase of cross-sectional area (CSA) of the median nerve at the forearm of 13.4 mm^2^ (**a**) and the dorsal roots C5 (upper diameter, 5.1 mm) and C6 (lower diameter, 4.4 mm) (**b**) at first presentation. After 2 months of therapy discontinuation, a significant reduction of CSA was visible in dorsal root C6 (**d**), which was consistent with a relevant clinical response of motor function and normalization of cranial nerves. After 5 months, the cross-sectional area of the median nerve (**c**) decreased from 13.4 to 8.4 mm^2^. Patient 2: NMUS showed nerve swelling of the vagus nerve with a CSA of 3.6mm^2^ (**e**), the increased diameter of the nerve roots C5 (3.78 mm) (**f**) and C6 (5.1 mm) (**h**) as well as the fibular nerve with an increased CSA of 11.6mm^2^ (**g**) at the onset of diffuse CNS and PNS symptoms only a few days after the 1st cycle of durvalumab. Abbreviations: *UA* upper arm

Based on the previously published data and our expertise in neuromuscular ultrasound of different neurological patient cohorts, we use NMUS as a supplementary technique in our neuro-oncological multimodal management of cancer patients presenting with suspected neurotoxicity. This non-invasive tool facilitates the differentiation of treatment-induced neuropathies from other causalities in the context of the overall clinical presentation. Here, we present two patient cases to exemplarily demonstrate the advantages and potential perspectives of the future NMUS applications and further cross-sectional research. Table 1 presents an overview of the clinical characteristics, and an overview of electrophysiological and NMUS findings (for more details see Additional file 1: Table S1); Fig. 1 shows relevant NMUS findings of both presented patients.

**Table 1:** Excerpt of clinical investigations and laboratory findings of presented cases

Investigations	Patient 1			Patient 2	
	Initial visit	Month 2	Month 5	Initial visit	Reference range
*Blood tests*					
CRP	8.5 mg/l	–	65 mg/l	444.2 mg/l	< 5.0 mg/l
IL-6	–	–	–	385 pg/ml	< 7.0 pg/ml
sIL-2 receptor	–	–	–	5611.0 U/ml	158–623 U/ml
Auto AB	Negative	–	–	Negative	Negative
*CSF analysis*					
Cell count	1 cells/µl	–	–	118 cells/µl	0–5 cells/µl
Protein	347 mg/l	–	–	964 mg/l	150–400 mg/l
Lactate	1.6 mmol/l	–	–	4.5 mmol/l	1.2–2.1 mmol/l
Glucose CSF/serum ratio	58.7%	–	–	–	> 50%
Virology	Negative	–	–	Negative	
Microbiology	Negative	–	–	Negative	
Cytology	–	–	–	93% neutrophil granulocytes 1% eosinophil granulocytes 1% monocytes 6% lymphocytes	
Neuro-pathology	No atypical cells	–	–	Signs of active inflammation, granulocytes dominating, no atypical cells	
CT staging	Partial response	–	Progressive disease	Progressive Disease	
Cerebral MRI	No evidence for leptomeningeal disease/CNS metastasis	No evidence for leptomeningeal disease or CNS metastasis	–	Diffuse contrast enhancement of the meninges	
*Neuro-graphy*					
Median nerve					
Sen	No potential bilaterally	No potential bilaterally	No potential bilaterally	No potential	Amplitude > 5.0 mV
Mot	No potential	Small amplitude (0.1 mV), reduced velocity (45.4 m/s); right arm	Small amplitude (0.3 mV/0.8 mV), reduced velocity (35.5/46.5 m/s); right/left arm	Normal	Velocity > 50.0 m/s
Tibial nerve	No potential	No potential	No potential	Decreased motoric amplitude (4.5 mV); right leg	Amplitude > 5.0 mV
Sural nerve	No potential	No potential	No potential	Decreased amplitude (3.1 µV); left leg	Amplitude > 3.8 µV
*NMUS findings*					
Median nerve	Almost continuous swelling (both sides, right > left)	Decrease of swelling (right)	No swelling (right), CTS (right)	Carpal-tunnel-syndrome (both sides)	
Ulnar nerve	Discontinuous swelling (both sides)	Decrease of swelling (right)	No swelling (right)	Swelling (right)	
Tibial nerve	n.a	Normal (left)	n.a	Tarsal-tunnel syndrome (both sides)	
Fibular nerve	n.a	Normal (left)	n.a	Swelling (both sides)	
Vagus nerve	Normal (both sides)	Normal (right)	n.a	Swelling (right)	
C5 longitudinal	Normal (both sides)	Normal (right)	n.a	Swelling (left)	
C6 longitudinal	Swelling (right)	Reduced swelling (right)	n.a	Swelling (left)	
Sural nerve	n.a	Normal (left)	n.a	Normal (both sides)	

*AB* antibodies, *n.a*. not applicable, *CRP* c-reactive protein, *IL-6* interleukin 6, *sIL-2* soluble interleukin 2 receptor, *CSF* cerebrospinal fluid, *CT* computer tomography, *NMUS* neuromuscular ultrasound, *MRI* magnet resonance imaging, *CNS* central nervous system

### Case 1

A 59 year old woman was diagnosed with metastatic breast cancer (lymph nodes, liver) and consecutively treated with several chemotherapies for ten months prior to first neurological presentation. She initially received palbociclib and developed a treatment related severe peripheral neuropathy III°, which improved after palbociclib termination. The symptoms aggravated after administration of paclitaxel. The therapy was then switched to eribulin, which the patient received about four weeks prior to first neurological presentation.

Palbociclib is a cyclin-dependent kinase 4 and 6 inhibitor, which obviates the phosphorylation of the retinoblastoma protein and inhibits cell-cycle progression from G1 to S phase [21]*.* Paclitaxel is a chemotherapeutic drug blocking cell cycle progression and preventing mitosis by promoting the assembly of tubulin into microtubules and preventing the dissociation of microtubules [22]. Eribulin’s cytotoxic effects are caused by inhibiting mictrotubule dynamics through suppression of microtubule depolymerisation [23].

She presented with progressive hypaesthesia in her legs, which had been present for about six months. For three months she had been noticing the same symptoms in her arms. Moreover, she developed difficulties closing her mouth and progressive hoarseness. At the time of admission to our neurological department, the patient showed a flaccid tetra paresis mainly involving her lower limbs, which caused a wheelchair-dependence and a serious reduction of activities of daily life. She presented with generalized muscle atrophy, severe hypaesthesia in the distal parts of her limbs, hoarseness, and facial palsy on both sides. The last course of chemotherapy had been administered a few days before.

The MRI of the brain did not show any pathology, especially no evidence of leptomeningeal spread or CNS metastasis. An extensive cerebrospinal fluid (CSF) analysis did not show any signs of inflammation or neoplastic affection. A CT of the throat and thorax gave no evidence for local metastasis in the larynx as a cause for her hoarseness. In conclusion, lymph node metastases and the primary tumor in the right mamma presented smaller than three months previously, which was in line with partial response to the current cancer treatment and therefore could not explain her clinical presentation.

In the first neurography, we saw a severe polyneuropathy with no motoric or sensory potentials in both median nerves, left tibial and sural nerves. Electromyography showed signs of acute denervation in the right vastus lateralis muscle. Due to the potential loss, no differentiation between axonal and demyelinating types of neuropathies was possible. At this time, NMUS revealed an almost continuous swelling of both median nerves (right > left) with fascicular enlargements as well as enlargement of the right C6 nerve root and discontinuous enlargement of both ulnar nerves. The nerves of the lower limbs were not examined initially due to compression stockings. The absence of evidence supporting concurrent conditions, combined with the connection to previously administered diverse neurotoxic agents, led us to the diagnosis of a severe treatment-related sensorimotor polyneuropathy with cranial nerve involvement. In agreement with the treating oncologist team the chemotherapy was discontinued. The patient received intensive physiotherapy and occupational therapy.

After eight weeks, the patient reported an improvement of general condition and the above-mentioned neurological symptoms. She was able to stand for a few minutes and to walk a few steps with a one-sided support. On neurological examination, the flaccid tetra paresis and paraesthesia of the hands and lower limbs were improving, the facial palsy, dysphagia and hoarseness were absent. Neurography still showed a severe polyneuropathy without electrical potentials in the left median nerve, tibial and sural nerve. There was a small motoric amplitude in the right median nerve. NMUS was performed again and showed a reduced CSA enlargement of the right median nerve and the cervical root.

About half a year after her initial presentation, the patient showed an unchanged tetraparesis but she reported newly gained sensation in her legs. Neurography revealed slow motoric amplitudes in both median nerves and left ulnar nerve without any sensory potential in the investigated nerves. NMUS showed regressive swelling without the previously observed evidence of nerve enlargements in the right median nerve and the right ulnar nerve. A severe carpal-tunnel-syndrome (CTS) was still present in the right median nerve (CSA 16.9mm^2^).

### Case 2

A 65 year old male patient had been diagnosed with a progressive cholangiocarcinoma with peritoneal carcinosis, lymph node metastasis and abdominal wall metastasis. He received the 1st cycle of a palliative cytostatic chemotherapy combined with the immune checkpoint inhibitor durvalumab. Durvalumab is a human monoclonal antibody against PD-L1 [24].

Only two days after treatment initiation, the oncologist team presented the patient to our neurological department due to an acute vigilance disturbance, global aphasia, disorientation, hemiparesis on the left side, oculomotor disturbance and vomiting. In addition, the patient presented a progressive renal failure (creatinine at admission 63 µmol/l [ref. 62–106 µmol/l] (glomerular filtration rate 98 ml/min [ref. > 90 ml/min]), at presentation 178 µmol/l (glomerular filtration rate 33.8 ml/min)), elevated inflammation parameters (C-reactive protein 444.2 mg/l [ref. < 5.0 mg/l] and procalcitonin 6.37 ng/ml [ref. < 0.50 ng/ml], increased cytokine levels including IL-6 385 pg/ml [ref. < 7.0 pg/ml], soluble IL-2 receptor 5166.0U/ml [ref. 158–623U/ml], increasing gamma GT 4.23 µmol/l*s [ref. 0.17–1.19 µmol/l*s] as well as altered thyroid enzymes (T4 9.46 pmol/l [ref. 12.00–22.00 pmol/l]; T3 < 1.50 pmol/l [ref. 3.10–6.80 pmol/l]). An emergency CT of the head did not reveal a stroke or intracranial haemorrhage. As part of our interdisciplinary diagnostic management approach, an MRI of the head was performed, which showed a discrete diffuse enhancement of the meninges without any further pathologies. Extended CSF analysis revealed an increased count of leucocytes (118/µl) with granulocytic dominance (93%), enhancement of overall protein with 964 mg/l [ref. 150–400 mg/l] and lactate 4.5 mmol/l [ref. 1.2–2.1 mmol/l]. Multiplex-PCR was negative for common neurotropic bacterial and viral pathogens. Nevertheless, a calculated antibiotic treatment with ceftriaxone, ampicillin and acyclovir was initiated. An additional or concurrent tumor lysis syndrome was considered but could not explain all of the symptoms. Electrophysiological findings involved a normal motoric neurography of the right median nerve including f-waves without any sensory potentials, a decrease in motoric amplitude of the right tibial nerve and a reduction of sensory amplitude of the left sural nerve, which was consistent with sensory-motor axonal polyneuropathy. NMUS revealed nerve swelling in the right vagus nerve, the nerve roots C5 and C6 on the left side, the peroneus nerve on both sides and the ulnar nerve on the right side. Furthermore, signs of a carpal-tunnel-syndrome and a tarsal-tunnel syndrome on both sides were detected.

Due to the direct link to the administered immunotherapy, the symptom dynamics, the clinical presentation as well as the typical constellation of inflammatory parameters and multiple organ affection, we diagnosed a systemic inflammation with CNS and PNS involvement induced by immune checkpoint inhibition. Considering and treating potential differential diagnoses at the same time, we initiated an immediate immunomodulatory therapy with methylprednisolone (up to 1000 mg per day for 5 days, followed by stepwise prednisolone tapering) according to the international recommendations. After one day, the patient was able to speak and sit at the bed side again. He recovered from the severe encephalitic syndrome and sensomotoric deficits within a few days, the renal function improved, and IL-6 decreased (initially 385.0 pg/ml, after methylprednisolone treatment 85.2 pg/ml).

## Discussion

Thus far, nerve enlargement identified by NMUS is mainly reported in the context of demyelinating polyneuropathies, such as immune-mediated neuropathies, while in axonal polyneuropathies focal or generalized nerve enlargement is an uncommon finding [25]. CIPN is characterized as (predominantly) axonal neuropathy mostly affecting sensory nerves [1].

In addition, we performed a systematic search in PubMed for relevant articles on NMUS and chemotherapy- or immunotherapy-induced polyneuropathy in humans. Articles that were published until March 2023 and written in English were encluded. We used the following search terms: “neuropathy”, “CIPN”,” ‘chemotherapy-induced peripheral neurotoxicity’, ‘toxic neuropathy’, ‘peripheral neuropathy’ and ‘neurotoxicity syndromes’ combined with “cytostatic”, “chemotherapy”, “immunotherapy”, “immune therapy”,”immune checkpoint inhibitor”, “immune checkpoint inhibition”, “checkpoint inhibition”, “checkpoint inhibitor” and “ultrasound”, “sonography”, “nerve sonography”, “nerve ultrasound”, “neuromuscular sonography”, “NMUS” and “HRUS”. There were no exclusions due to article type. We identified 6 relevant publications (1 case report, 1 review, 1 retrospective study, 3 prospective studies) reporting on cancer patients who were treated with cytostatic agents. No publications regarding NMUS use for neuropathies induced by immunotherapies were identified. On account of the limited number of publications and patients included, a meta-analysis was not feasible. The basic characteristics of the analysis are summarized in Table 2.

**Table 2:** Literature overview for published articles addressing NMUS and chemotherapy/immunotherapy induced polyneuropathy (publications included until March 2023)

References	Publication/study type	No. of pat	Type of cancer	Treatment regimen	Control	Ultrasound findings
Portland et al. [26]	Case report	1	Breast cancer	Paclitaxel	No	NMUS performed of both median nerves only
						Enlargement of median nerve CSA at the wrists and palms, bilaterally
						Hypoechogenic nerves
						Reduced mobility of nerves
Erdmann et al. [27]	Monocentric, retrospective study	27*	n.a	Platinum based regimen	19 pat. with CIP + 20 pat. with CIDP	No CSA analysis described, focused on echogenicity only
						No significant difference described (tendency to slightly hyperechogenic findings)
Lycan et al. [28]	Monocentric, prospective cross-sectional study	20	Breast cancer	Taxane	healthy historical controls	decreased sural nerve size increased median nerve size, but on compression site only
Alberti et al. [29]	Review	n.a	n.a	n.a	n.a	n.a
Pitarokoili et al. [30]	Monocentric, prospective study	13	mPC	oxaliplatine, irinotecan and 5-fluorouracil (FOLFIRI-NOX)	No	Tendency for CSA increase at compression sites for ulnar nerve (elbow) and radial nerve (radial groove) increased CSA for fibular nerve (at fibular head; 10/13) and the tibial nerve (at knee and ankle)
Briani et al. [31]	Monocentric, prospective study	15	CRC	Oxaliplatine	No	CSA increase of median and ulnar nerves at compression sites (9/15), in 4/15 pat. bilaterally

*CIDP* chronic inflammatory demyelinating polyneuropathy, *CIP* critical illness polyneuropathy, *CRC* colorectal cancer, *CSA* cross sectional area, *n.a.* not applicable, *NMUS* neuromuscular ultrasound, *mPC* metastatic pancreatic cancer, *pat.* patients

^*^13/27 patients already described at Pitarokoili et al. [30]

As shown in our systematic literature search we identified a case report as well as a few monocentric retrospective and prospective studies showing CSA increase of multiple nerves in patients with chemotherapy-induced polyneuropathy. This is consistent with our clinical experience and the case studies presented in this work.

In patient 1, atypical presentation of cranial nerve involvement and a severe affection of motoric functions led to extensive diagnostics to exclude leptomeningeal disease, infectious and autoimmune CNS/PNS affection of other causality. After excluding concurrent conditions and based on the additional NMUS findings, an interdisciplinary consent led to discontinuation of epirubilin. The increased CSA of the cervical dorsal roots and the pattern of continuously enlarged multiple nerves did not fit any known constellation, e.g., CIDP. This supported the hypothesis of therapy-induced neuropathy, potentially with an accompanying inflammatory component as described in some previously published cases of CIPN. Neither electroneurography nor CSF and extensive blood testing were diacritic to confirm neuropathy induced by cancer treatment. Patient 1 exhibited a significant reduction of the neurological symptom burden after only a few weeks of treatment interruption. At the same time, NMUS pathologies improved stepwise (without any concurrent therapeutic intervention besides physiotherapy/occupational therapy). In this case, NMUS has proven to be a very helpful tool for both the diagnosis and neuromonitoring of neurotoxicity compared with neurography findings, which showed improvement in a delayed fashion. This case likewise illustrates the chance of symptom improvement even in cases of severe neuropathies within an interdisciplinary expert setting.

In patient 2, the additional NMUS assessment showed a strong PNS inflammation where a severe CNS inflammation syndrome was dominating the clinical decision making. As the patient was unable to cooperate in a sufficient manner or describe the subjective sensorimotor symptoms initially, NMUS contributed to differentiate immune-related inflammation of the nervous system from other conditions. Therefore, the technique seems to be helpful in distinguishing between peripheral and central causes of muscular weakness in cases of impaired communication. According to this, the value of NMUS as a specific biomarker of peripheral nirAEs should be investigated in future prospective studies.

In our patients, neuromuscular ultrasound revealed increased CSA in several nerves with partly continuous and partly discontinuous swelling, which is consistent with a major part of the reviewed studies. Moreover, in both patients the CSA of the dorsal roots and the vagus nerve in patient 2 were enlarged, which has not been reported in treatment-induced neuropathy so far. Both patient cases show the importance of extensive clinical and paraclinical neurological assessment in case of newly occurring neurological symptoms in systemically treated cancer patients. Furthermore, we emphasize the role of specialized neurologists or neuro-oncologists in this extremely interdisciplinary field for decision making in cases with such atypical clinical constellations of suspected neurotoxicity.

All previous studies, however, recruited patients with subjective suffering from neuropathy symptoms. Thus, it is currently not possible to discuss NMUS findings in cancer patients receiving neurotoxic agents but without relevant neurological symptoms. Most identified studies reported CSA increase of median nerves. Further nerve swelling was described in ulnar, radial, tibial and fibular nerves mostly presenting as concurrent enlargement of multiple nerve sites. Portland et al. performed a NMUS of both median nerves only, so a statement regarding the general swelling pattern in this patient case is not possible [26]. A potentially increased sensitivity to treatment-induced neuropathies in cancer patients with pre-existing compression syndromes such as carpal-tunnel-syndrome should be considered. All identified studies contained small groups of patients (< 30 patients each). Therefore, only descriptive statistical analysis was possible. The study designs regarding measured parameters and control groups were heterogeneous impeding a comparison and generalization of the presented data. Historically, healthy controls and CSA analysis were used in the prospective study of Lycan et al., whereas Erdmann et al. compared CIPN with CIDP and critical illness neuropathy patients without a healthy control cohort and regarding nerve echogenicity only without CSA assessment data presented in the manuscript [27, 28]. Moreover, we had difficulties retrieving information about the quantitative NMUS scores being used for the single cohorts in the studies.

A standardized neurological assessment of cancer patients with solid or haematological diseases who develop new neurological symptoms during or shortly after the treatment with neurotoxic agents is essential for early recognition of treatment-associated effects. To date, there is no effective preventive procedure for CIPN or immune-related neuropathies although interventions regarding dietary recommendations, medication and exercise are currently under investigation [32]. Early identification and neuromonitoring may lead to sufficient symptom control, more personalized cancer treatment and dose adjustment while avoiding long-term quality of life impairment [33, 34]. Moreover, the knowledge of clinical patterns of neurotoxicity is the key for the challenging differentiation between treatment-induced neuropathies, paraproteinemic conditions, leptomeningeal spread mimicking a polyradiculitis as well as “classical” neurological diseases (amyotrophic lateral sclerosis (ALS), multiple sclerosis MS, GBS/CIDP) in cases with atypical clinical presentation. In addition, there is an urgent need for valid biomarkers and pain-free, cost-effective techniques to differentiate treatment-induced neuropathy from other types of neuropathies and to monitor the severity as well as treatment response in terms of modern combinations of cytostatic and immunotherapeutic agents.

**Fig. 2:**
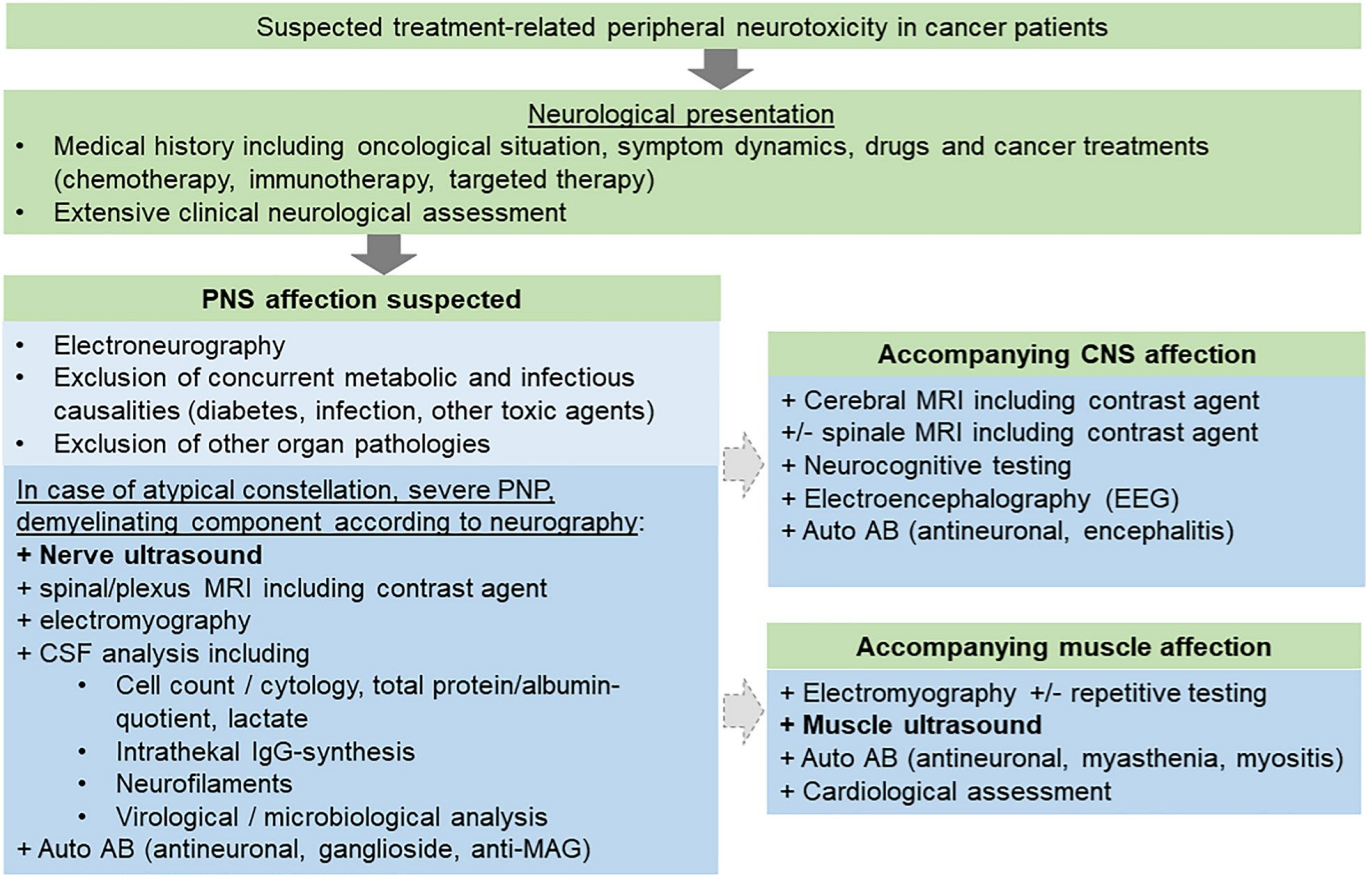
Diagnostic algorithm for cancer patients presenting with suspected treatment-related peripheral neurotoxicity. The figure shows our propose for a standardized diagnostic algorithm including NMUS as a part of routine neurological assessment for cancer patients with suspected peripheral neurotoxicity. Abbreviations: *CNS* central nervous system, *CSF* cerebrospinal fluid, *MRI* magnet resonance imaging, *IgG* immune globulin G, *Auto AB* auto antibodies, *anti MAG* antibodies against myelin-associated glycoprotein, *PNP* polyneuropathy, *PNS* peripheral nervous system

In summary, this is the first description of enlargements of dorsal roots and vagus nerve in cancer patients with treatment-induced neuropathies. In addition, we present a diagnostic algorithm that incorporates NMUS (as shown in Fig. 2) as an integral aspect of the neurological assessment for cancer patients. The algorithm may be particularily valuable as a part of the routine neurological assessment for cancer patients with suspected peripheral neurotoxicity in case of atypical presentations, severe symptoms, or acute onset, aiding in the process of differential diagnosis.

However, this current work shows representative examples from interdisciplinary NMUS integrative work-up only and does not allow any generalization of the identified NMUS findings. Taken together with previous studies, our experience nevertheless supports the promising potential of NMUS in this field and the importance of prospectively designed, cross-sectional studies for cancer patients with newly occurring neuropathies.

## Conclusion

At our institution, neuromuscular ultrasound is an integral part of the interdisciplinary algorithm for peripheral neuropathies in systemically treated cancer patients. NMUS is a suitable technique for longitudinal neuromonitoring. Neuromuscular NMUS should be validated with regard to potentially specific patterns of nerve enlargement caused by cytostatic agents and immunotherapies. Moreover, valid control groups with known patterns of nerve enlargement such as CIDP or GBS as well as healthy controls are crucial to define the relevance of neuromuscular ultrasound in this exceptionally interdisciplinary field. In conclusion, NMUS could expand the neurological tool inventory in differential diagnosis of cancer patients in a non-invasive and cost-sparing manner. Prospective studies should define the diagnostic relevance of NMUS for patients treated with agents potentially affecting the PNS and its impact on cancer patient care.

### Supplementary Information

**Additional file 1. Table S1**: Detailed NMUS measurements of the presented patients.

## Data Availability

The dataset presented in this study are available on reasonable request from the corresponding author.

## Abbreviations

AB: Antibodies
ALS: Amyotrophic lateral sclerosis
Anti MAG: Antibodies against myelin-associated glycoprotein
Auto AB: Auto antibodies
CIDP: Chronic inflammatory demyelinating polyneuropathy
CIP: Critical illness polyneuropathy
CIPN: Chemotherapy-induced peripheral neuropathy
CMAPs: Compound muscle action potentials
CNS: Central nervous system
CRC: Colorectal cancer
CRP: C-Reactive protein
CSA: Cross sectional area
CSF: Cerebrospinal fluid
CT: Computer tomography
CTS: Carpal-tunnel-syndrome
DNA: Deoxyribonucleic acid
GBS: Guillain–Barré syndrome
HRUS: High-resolution ultrasound
IL-2/IL-6: Interleukin 2/6
IgG: Immune globulin G
irAEs: Immune-related adverse events
MMN: Multifocal motor neuropathy
mPC: Metastatic pancreatic cancer
MRI: Magnet resonance imaging
MS: Multiple sclerosis
n.a.: Not applicable
NF1: Neurofibromatosis type 1
nf2: Neurofibromatosis type 2 gene
NF2: Neurofibromatosis type 2
nirAEs: Neurological irAEs
NMUS: Neuromuscular ultrasound
pat.: Patients
PD-L1: Programmed-cell death-ligand 1
PNP: Polyneuropathy
PNS: Peripheral nervous system
POCUS: Point of care ultrasound
sIL-2: Soluble interleukin 2 receptor
SNAP: Sensory nerve action potentials
UPSS: Ultrasound Pattern Sum Score
