# Supplementary material for: The role of neuromuscular ultrasound in diagnostics of peripheral neuropathies induced by cytostatic agents or immunotherapies
Source: Acta Neuropathol Commun. 2023 Nov 27;11:187. doi: 10.1186/s40478-023-01685-9 (PMC10683078; doi:10.1186/s40478-023-01685-9)
Supplement: Supplementary file 1 — Additional file 1. Table S1: Detailed NMUS measurements of the presented patients. [file 40478_2023_1685_MOESM1_ESM.docx]

**Supplementary Table 1: Detailed NMUS measurements of the presented patients.**

|  | | **Patient 1** | | | **Patient 2** |  |
| --- | --- | --- | --- | --- | --- | --- |
| **Sensorimotor nerves** | | | | | | |
| **Measured nerve location** | | **CSA in mm^2^** | | | | **Normal**  **<50%**  **>50%** |
| Median nerve | Upper arm | R: 13.6  L: 13.1 | R: 10.8-12.9  L: - | R: 8.4  L: - | R: 9.03  L: - | <12  ≥12 ≤18  >18 |
|  | Elbow | R: 12.6  L: 12.6 | R: 9.5  L: - | R: 7.1  L: - | R: 7.86  L: - | <12  ≥12 ≤18  >18 |
|  | Forearm | R: 12.0  L: 9.9 | R: 9.2-11.0  L: - | R: 5.9  L: - | R: 7.27  L: 6.9 | <10  ≥10 ≤15  >15 |
|  | Wrist | R: 17.8  L: 14.5 | R: 15.9  L: - | R: 16.9  L: - | R: 15.81  L: 15.0 |  |
| Ulnar nerve | Upper arm | R: 9.9  L: 7.9 | R: 8.4  L: - | R: 6.8  L: - | R: 10.05  L: - | <9.5  ≥9.5 ≤14.25  > 14.25 |
|  | Elbow | R: 12.2  L: 11.3 | R: 12.8  L: - | R: 9.6  L: - | R: 7.16  L: - |  |
|  | Forearm | R: 8.2  L: 9.0 | R: 6.9  L: - | R: 4.25 + 1.4  L: - | R: 8.0  L: 6.2 | <8.5  ≥8.5 ≤12.75  >12.75 |
|  | Wrist | R: 5.9  L: - | R: 6.8  L: - | R: 2.8 + 0.8  L: - | R: 4.71  L: 6.2 |  |
| Tibial nerve | Popliteal | R: -  L: - | R: -  L: 20.8 | R : -  L : - | R: 25.8  L: 20.3 | <33  ≥33 ≤49.5  >49.5 |
|  | Ankle | R: -  L: - | R: -  L: 12.5 | R : -  L : - | R: 16.6  L: 18.3 | <14  ≥14 ≤21  >21 |
| Fibular nerve | Popliteal | R: -  L: - | R: -  L: 6.1 | R : -  L : - | R: 11.6  L: 12.4 | <11.5  ≥11.5 ≤17.25  >17.25 |
| **Sensory nerves** | | | | | | |
| **Measured nerve location** | | **CSA in mm^2^** | | | | **Reference range** |
| Sural nerve | Calf | R: 3.4  L: 2.5 | R: -  L: 2.4 | R : -  L : - | R : -  L : - | ≤ 3.5 |
| Superficial radial nerve |  | R : 1.6  L : - | R : -  L : - | R : -  L : - | R : -  L : - | ≤3.0 |
| Superficial peroneal nerve |  | R : 1.2  L : 0.8 + 0.8 | R : -  L : - | R : -  L : - | R : -  L : - | ≤3.5 |
| **Cervical roots and vagus nerve** | | | | | | |
| **Measured nerve location** | | **CSA in mm^2^** | | | |  |
| Vagus nerve | Carotid sheath | R: 2.0  L: 1.4 | R: 1.4  L : - | R : -  L : - | R: 3.66  L: 1.88 | ≤3.5 |
| **Measured nerve location** | | **Diameter in mm** | | | |  |
| C5 longitudinal | Transversal process | R: 2.5  L: 2.5 | R: 2.7  L: - | R : -  L : - | R: 2.87  L: 3.78 | ≤ 2.9 |
| C6 longitudinal | Transversal process | R: 5.1  L: 4.1 | R: 4.4  L: - | R : -  L : - | R: 3.7  L: 5.13 | ≤ 4.2 |
